# Aperture-Controlled Fabrication of All-Dielectric Structural Color Pixels

**DOI:** 10.1021/acsami.3c03353

**Published:** 2023-06-29

**Authors:** Clémentine Lipp, Audrey Jacquillat, Daniel Migliozzi, Hsiang-Chu Wang, Arnaud Bertsch, Evgenii Glushkov, Olivier J.F. Martin, Philippe Renaud

**Affiliations:** † École Polytechnique Fédérale de Lausanne EPFL-STI-IMT-LMIS4, Station 17, Lausanne CH-1015, Switzerland; ‡ École Polytechnique Fédérale de Lausanne EPFL-STI-IMT-NAM, Station 11, Lausanne CH-1015, Switzerland

**Keywords:** structural colors, thin films, interference, micropatterns, single-mask process

## Abstract

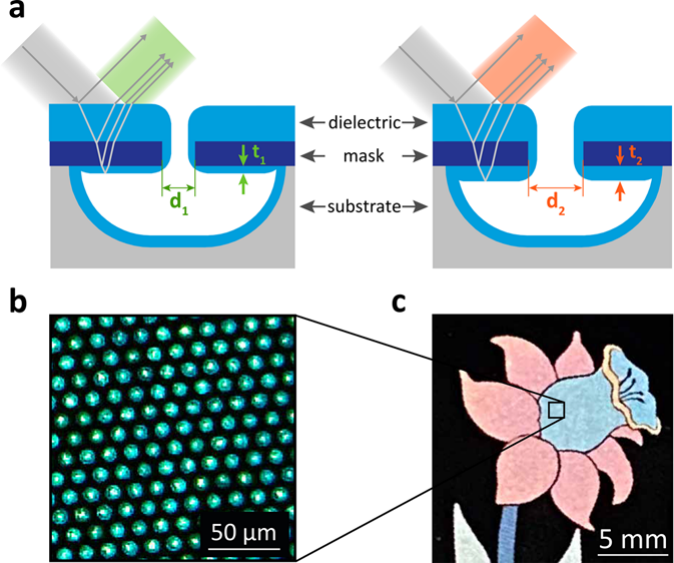

While interference colors have been known for a long time, conventional color filters have large spatial dimensions and cannot be used to create compact pixelized color pictures. Here we report a simple yet elegant interference-based method of creating microscopic structural color pixels using a single-mask process using standard UV photolithography on an all-dielectric substrate. The technology makes use of the varied aperture-controlled physical deposition rate of low-temperature silicon dioxide inside a hollow cavity to create a thin-film stack with the controlled bottom layer thickness. The stack defines which wavelengths of the reflected light interfere constructively, and thus the cavities act as micrometer-scale pixels of a predefined color. Combinations of such pixels produce vibrant colorful pictures visible to the naked eye. Being fully CMOS-compatible, wafer-scale, and not requiring costly electron-beam lithography, such a method paves the way toward large scale applications of structural colors in commercial products.

## Introduction

Since ancient times people have tried to reproduce the colors they saw in nature by using, first, natural pigments and, later, synthetic dyes.^1^ The color of these materials is produced thanks to the absorption of specific wavelengths of incident white light and the reflection of others. A different mechanism of color generation comes in play when incident light is not absorbed, but rather deflected by a material in a certain way due to the presence of an internal structure with dimensions of the same order as the light wavelength. Such ”structural” colors have been first described in the 17th century, based on the observations of the iridescent peacock feathers.^2,3^ The first visual evidence of the complex microscopic structure of these feathers came two and a half centuries later with the invention of the scanning electron microscope,^4^ which became the main tool for studying structural coloration in nature.^5,6^

The potential of bioinspired structural colors featuring higher stability and lower photodegradation, while being more environmentally friendly compared to traditional dyes, was clear. However, fully harnessing this potential in man-made materials only became possible with the advent of micro- and nanostructuration tools.^7,8^ One of the first platforms to demonstrate the generation of artificial structural colors were metallic nanoantennas utilizing localized surface plasmon resonances.^9^ Fabricated either using electron beam lithography (EBL) or pulsed femtosecond lasers, they were able to provide an unprecedented printing resolution of structural color pixels up to >10^5^ dots per inch (DPI)^10,11^ on various materials.^12−14^ Such structural colors have found numerous applications in several areas, ranging from solar energy harvesting^15^ to displays,^16^ security and anticounterfeiting.^17−20^

The downside of using metals for the implementation of structural colors is not only their high cost but also inherent optical losses, limiting the generation of saturated colors from the wide gamut available for traditional dyes. In contrast to plasmonic nanostructures, dielectric metasurfaces have much lower optical losses and, therefore, provide an efficient way of generating bright structural colors,^21−25^ but still rely on focused ion beam (FIB) or EBL techniques to define the individual color pixels. The inherent dependence on these tools leads to high manufacturing costs and severely limits the scalability and industrial applications of such structural colors.^26,27^

**Figure 1:**
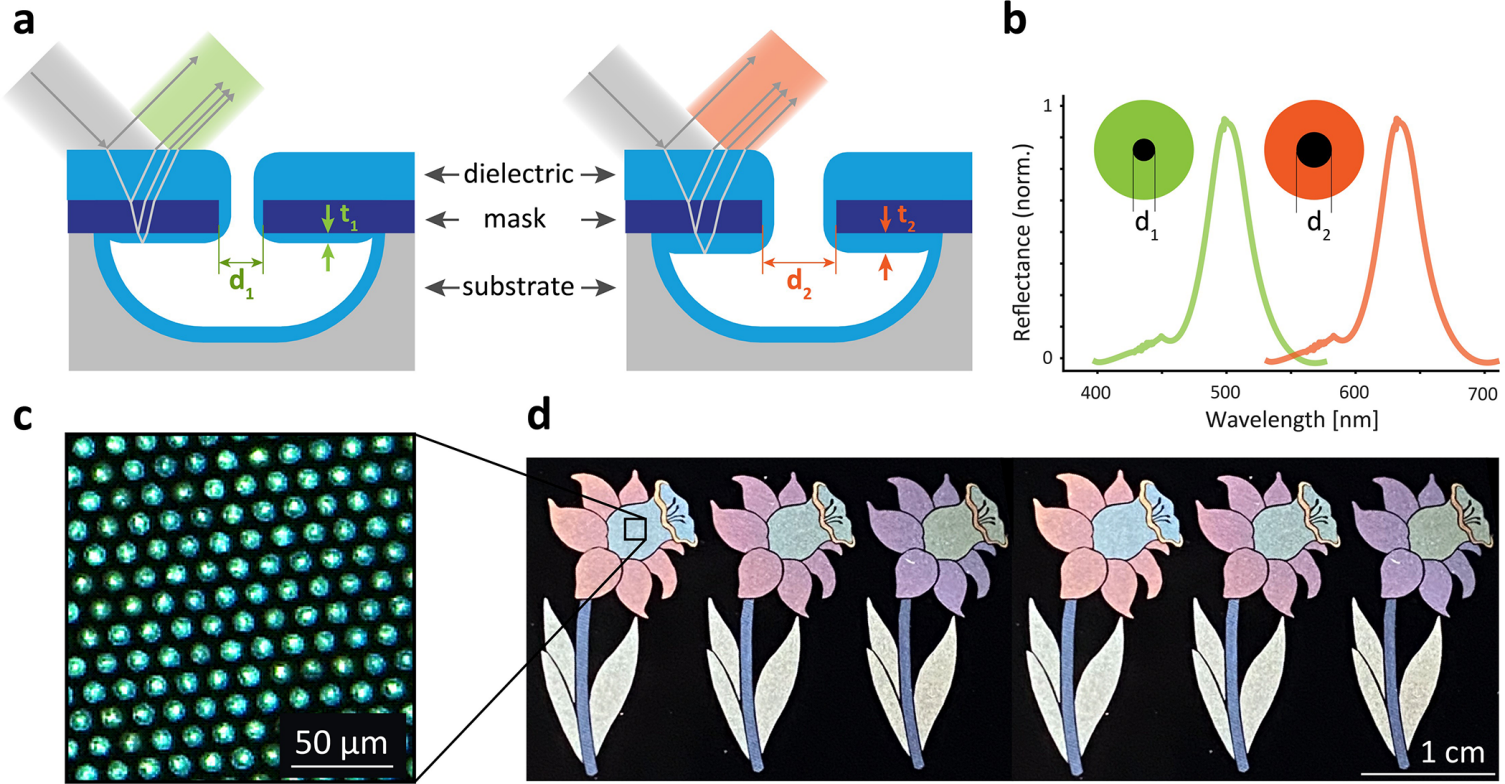
Concept of the aperture-controlled thin film stack for the interference-based color pixels. (a) Side view: a cavity is formed via uniform etching of the substrate through a circular aperture of a respective diameter (*d*_1_ and *d*_2_). A subsequent deposition of another dielectric creates a three-layer thin film stack with an aperture-controlled thickness of the bottom layer, deposited inside the cavity (*t*_1_ and *t*_2_). The thickness of the top two layers stays the same regardless of the size of the aperture, while the bottom layer has a variable thickness, depending on the diameter of the opening, which leads to a difference in the spectra of reflected light. (b) Top view: the reflectance spectrum and, therefore, the perceived color of the cavity are thus controlled by the aperture. (c) Each cavity can be used as a pixel of an individually controlled color. (d) Such pixels can be arranged in patterns to form structurally colored images.

In this context, we propose a novel method to locally control the color, produced by a stack of thin dielectric films^28^ using only a single UV photolithography step. The photolithography is used to define the openings in the dielectric mask, which in turn influence the deposition of a second dielectric layer inside the created cavities (see Figure 1). Such an aperture-controlled deposition allows us to control the constructive interference of the incident and the reflected light, thus turning the cavities into micrometer-scale color pixels. Combining such pixels we can produce millimeter- to centimeter-scale color pictures visible to the naked eye. The whole fabrication process, described in the following sections, is fully CMOS-compatible, and can be easily scaled up using existing semiconductor foundries, which makes it very attractive for large-scale use in commercial products. We therefore believe that it will likely be adopted by industry, finding numerous applications from anticounterfeiting elements to iridescent surface decorations in high-end watches and jewelry.

## Results

The proposed method of creating structural color pixels is conceptually shown in Figure 1a. The local color is produced by the incident white light interfering at the interface of a fabricated thin film stack, where the thickness of the bottom layer is controlled by the nonconformal deposition of a dielectric inside a preformed hollow cavity.^29,30^ The cavity itself is etched isotropically in the substrate underneath a transparent mask featuring an opening of a designed diameter, defining a circular region underneath the suspended mask. After the cavity is formed, a second type of dielectric, different from that forming the mask, is deposited in conditions tailored for a nonconformal coating of the inner surfaces.

In particular, its thickness inside the cavity underneath the mask is limited and controlled by the diameter of the hole in the mask. The fabricated structure seen from the top represents a circle and is composed by a suspended stack of three layers with two constant thicknesses, the top and middle layers, and a variable bottom one. This stack of thin films forms an optical interface and due to interference of the incident and the reflected light the spectrum of the reflected light is specific to the stack, resulting in particular visible colors. The described principle of an aperture-controlled stack of dielectrics and its specific reflectance spectrum is illustrated in Figure 1a, b. The holes in the mask with surrounding cavities and thin film stacks creating specific colors can be arranged in a pattern to define a colored picture, similar to pixels arranged to form an image. An example of an image formed by these structures is shown in Figure 1d with the inset image Figure 1c detailing the pixels.

### Fabrication

**Figure 2:**
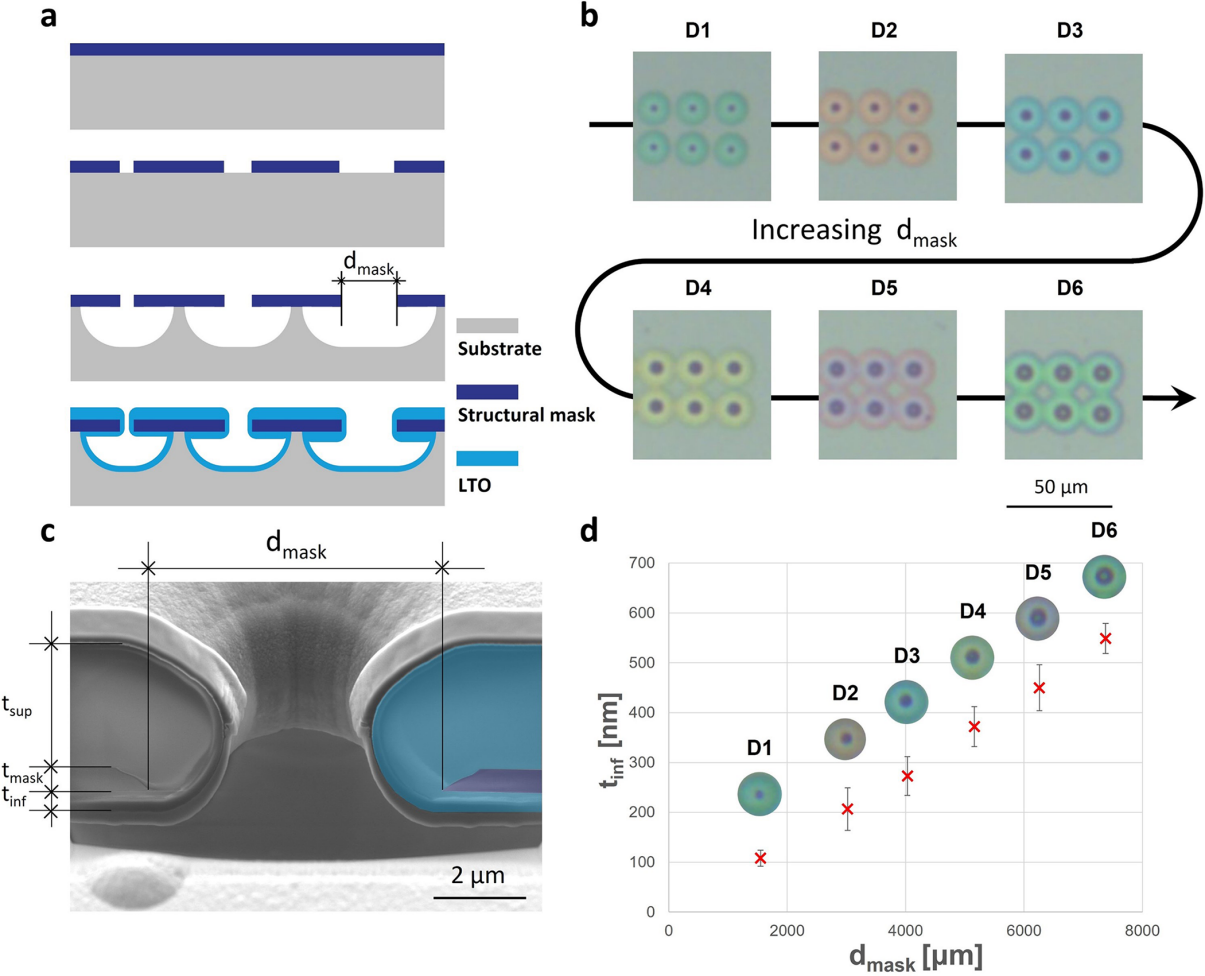
(a) Side view of the fabrication process used to create the color pixels: (i) a thin transparent mask is deposited on a substrate and (ii) patterned with circular apertures of different diameters *d*_mask_. (iii) The substrate is selectively and isotropically etched, producing cavities with an overhanging mask. (iv) A deposition of LTO is then performed, which has the property of depositing non conformally. The thickness of LTO under the overhanging mask is thus defined by the value of *d*_mask_. (b) Top view of the six test structures fabricated using the proposed process with fused silica as a substrate and Al_2_O_3_ as a mask. *d*_mask_ ranges between 1 and 8 μm, leading to the creation of different colors. (c) FIB cut of a fabricated structure revealing the overhanging thin film stack. The LTO layer is colored in blue and the Al_2_O_3_ in purple, for better visualization, and two extracted parameters are indicated. (d) Measured bottom LTO layer thickness *t*_inf_ as a function of the aperture in the mask for the same six test structures shows a linear dependence.

The fabrication process to create structural color pixels is illustrated in Figure 2a and starts with the deposition of the mask material onto a substrate wafer. The choice of the mask material is dictated by its optical properties (transparency to light of a chosen wavelength) and etch rate selectivity relative to the substrate material to ensure the efficient cavity etching process (e.g., plasma, gas or wet etch). The openings of diameter *d*_mask_ are then defined by UV photolithography and transferred onto the mask using an adapted etching process (see Materials and Methods). Third, the substrate is selectively and isotropically etched to form the cavities with the suspended mask on top. The spacing between the openings combined with the lateral dimensions of the under-etch can be tailored to lead to two different outcomes: when the spacing between the openings is smaller than the diameter of the cavity, the cavities merge due to the under-etch and form a larger area of the suspended mask material. This option can be interesting to increase the fill factor, but can be problematic because of the stress in the thin film and will not be explored in detail here. However, if the spacing is chosen to be larger than the diameter of the cavities, the mask layer is regularly anchored to the substrate which mitigates stress issues. Therefore, this regime will be primarily used for the purpose of the work presented here (Figure 2b).

The next fabrication step is the controlled deposition of a second dielectric with a refractive index different from that of the mask material. The deposited film has to be nonconformal to the shape of the cavity, so that its thickness at the backside of the etched mask depends on the size of the opening. One well-known method for such nonconformal deposition of a dielectric is the low-pressure chemical vapor deposition (LPCVD) of low-temperature silicon dioxide (LTO). LTO is deposited at low pressure (150 mTorr here) and low temperature (425 °C) compared to other LPCVD processes. The surface coverage of LTO on similar structures was studied by Cheng et al.,^29^ who deduced that the material deposited inside the cavity underneath the overhang is due to re-emission of the precursor owing to its low sticking coefficient, and not due to the surface diffusion of the deposited material. The generally accepted mechanism is that precursors with mean free paths much larger than the structure size deposit at the bottom of the cavity under the opening and are re-emitted underneath the overhang.^29^ Thus, the larger the opening in the mask (*d*_mask_) is, the larger is the amount of re-emitted material. Another parameter affecting the deposition under the overhang is the aspect ratio of the cavity: as the center-bottom part of the cavity acts as a point source, the larger the cavity’s width/height ratio, the more tapered is the resulting thin film profile. In our case, this aspect ratio is constant owing to the isotropic nature of the etching process and, when comparing to the test structures from Cheng et al.,^29^ we estimate the variation in thin film thickness under the overhang to be less than 15%. Specifically, the structures fabricated for this work were made on fused silica and Si substrates, using Al_2_O_3_ and Si_*x*_N_*y*_ as mask materials (the detailed fabrications process is provided in Materials and Methods). LTO was chosen as a second dielectric with a nominal film thickness over the mask layer set to 2.7 μm. The choice of materials was not only supported by their optical properties, but also by their ease of integration into CMOS-compatible industrial microfabrication processes.

### Cross-Section Characterization

In order to validate the described concept, test structures referred to as D1 to D6 were fabricated on a fused silica substrate with 460 nm thick Al_2_O_3_ film as a mask. Color pixels with *d*_mask_ between 1 and 8 μm were fabricated and are shown in Figure 2b: the black dot in the center of the pixel corresponds to the empty opening in the mask through which the under-etch was performed and the colored part indicates the presence of the overhanging thin-film stack causing interference in the reflected light. FIB cuts were performed on the six test structures to observe the cross section along their center. Figure 2c shows a scanning electron microscopy (SEM) image of such cut along with the parameters extracted from it: the diameter of the opening in the mask is referred to as *d*_mask_, the top LTO thickness as *t*_sup_, the middle Al_2_O_3_ thickness as *t*_mask_, and the bottom LTO thickness as *t*_inf_. The different materials are false-colored for an easier visual perception on the right part of the cut with the LTO layer in blue and the Al_2_O_3_ mask in purple. The nonconformal deposition is clearly visible with a *t*_sup_ being much larger than the *t*_inf_ value. A plot of the bottom thickness *t*_inf_ as a function of the hole diameter *d*_mask_ extracted from the FIB cuts is shown in Figure 2d, indicating a seemingly linear dependence of the bottom layer as a function of *d*_mask_ and validating the concept of an aperture-controlled thin film stack.

### Physical Modeling

To validate the physical phenomenon responsible for the appearance of colors in the regions surrounding the microapertures, we used the transfer matrix method^31^ to calculate the reflectance spectrum of the stack of thin layers and converted it to the CIE-1931 XYZ color space.

For the first step, we considered p-polarized light at normal incidence on the stack, and modeled the *j*th layer as a matrix:
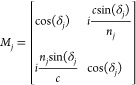
1where *i* is the imaginary unit, *c* is the speed of light, and δ_*j*_ is the phase shift induced on a plane wave of wavelength λ by the layer having refractive index *n*_*j*_ and thickness *t*_*j*_ defined as
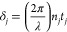
2

The total transfer matrix is then given by the product of the transfer matrices of the *L* layers in the order in which the light encounters the layers:
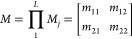
3

Finally, the reflectance spectrum is given by
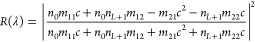
4

Where *n*_0_ and *n*_*L*+1_ are the refractive indices of the media preceding and following the stack, respectively.

To convert the reflectance spectrum into the color appearance under an illuminant with spectrum *I*(λ), we used the color matching functions *x*(λ), *y*(λ), and *z*(λ) to obtain the CIE-1931 XYZ color space as
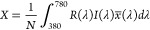
5
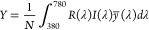
6
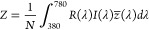
7with
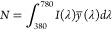
8

**Figure 3:**
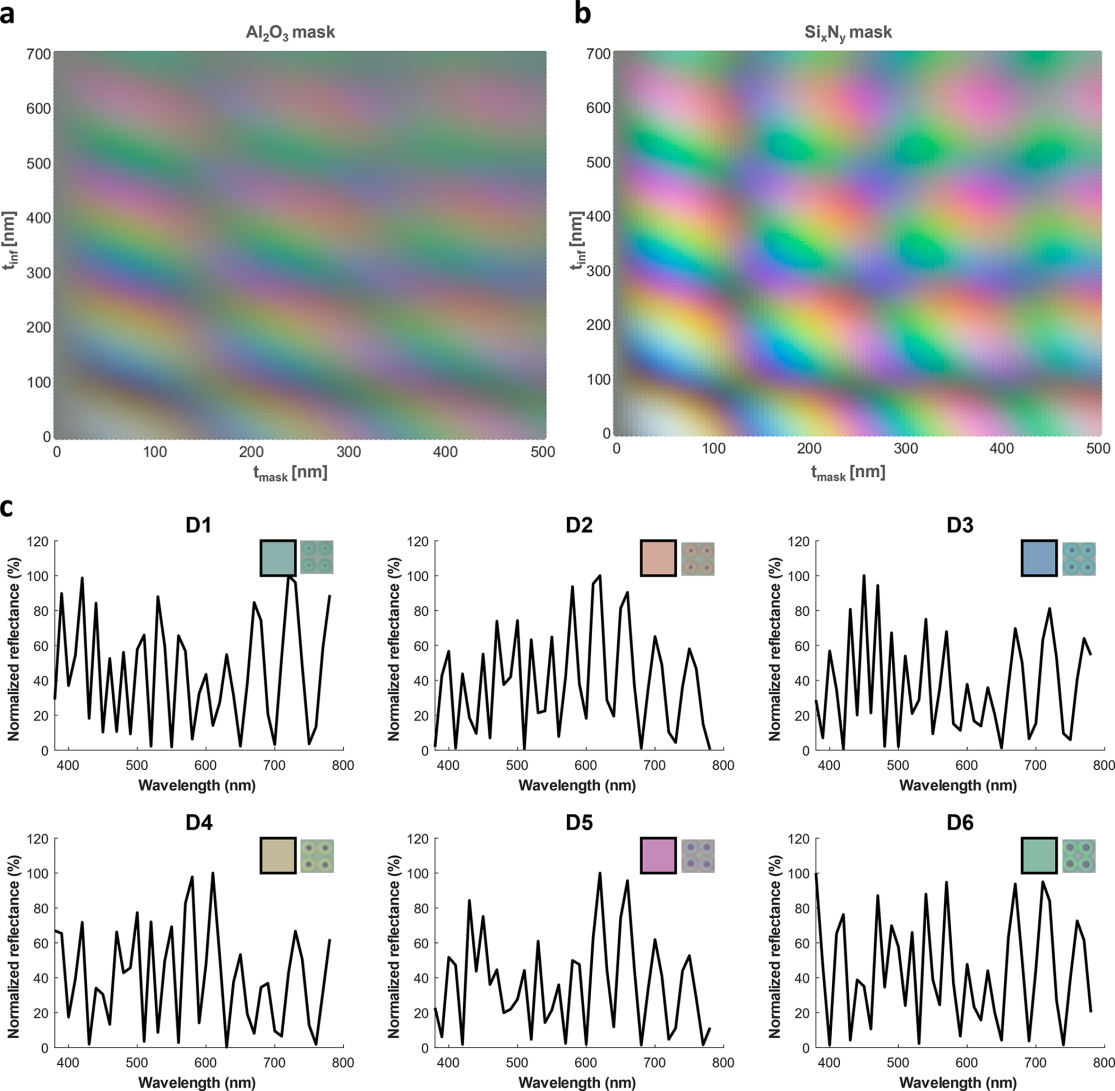
(a, b) Color appearance modeled with the transfer matrix method and the CIE 1931 XYZ color space for *t*_sup_ = 2700 nm and varying *t*_mask_ and *t*_inf_, for two different materials as a mask layer. The difference in color is due to the different refractive index of the two materials. (c) Predicted normalized reflectance spectra of the test structures with Al_2_O_3_ mask. Square insets display the predicted (rigid outline) and observed (dashed outline) color appearance. The theoretical spectrum was calculated based on the measured values: *t*_sup_ = 2700 nm, *t*_mask_ = 460 nm, and *t*_inf_ = 95, 205, 310, 370, 440, and 520 nm.

The spectrum of the incident light was measured from a halogen lamp source, while the colors of the reflected light were simulated by sweeping over the thicknesses of the three layers, *t*_sup_, *t*_mask_, and *t*_inf_, to generate a 3D volume with a corresponding color. As a result of such parameter sweep, a small variation (in the order of hundreds of nanometers) in the top SiO_2_ layer’s thickness was found to play an insignificant role in the generated color and was thus fixed to its measured value of 2700 nm. The simulated color palette for a sweep of two remaining parameters (*t*_mask_ and *t*_inf_) is represented in Figure 3a with Al_2_O_3_ as mask material. Here we observe a periodicity in both directions with more well-defined colors in the lower range of *t*_inf_ and *t*_mask_. The colors of the test samples represented in Figure 1b seem to match the color found on the color palette at the corresponding dimensions reported in Figure 1d. Figure 3b represents the color palette modeled in the same conditions for a mask made of a higher refractive index material, silicon nitride. The resulting shorter period in the *x* direction shows the expected influence of the larger refractive index of Si_*x*_N_*y*_ (*n* ≈ 2.4) versus Al_2_O_3_ (*n* ≈ 1.9). The simulated colors with the Si_*x*_N_*y*_ as the mask material are also more saturated than for Al_2_O_3_ and are thus promising for obtaining wide-gamut color palettes.

### Validation of Reflectance Spectra and Color Appearance

The interesting aspect of the current model is the ability to make predictions on the expected color appearance depending on the diameter of the hole. By using the SEM measurements of Figure 2d, we compared the predicted color appearance with the observed colors of the microfabricated test structures (Figure 3c). To account for reflectance and illuminance variations due to real measurement conditions, we normalized the simulated reflectance spectra to have maximum brightness: this exalts the tone and make the colors very visible while preserving the ratios of the contribution for each wavelength. Remarkably, we found that the accordance between the predicted and the observed color is very high for all the apertures tested. By using the raw spectra depicted in Figure S1, the outcomes are not qualitatively different: the predicted spectra follow the measured ones, and colors remain the same (*i.e*., a red shade is reddish, a green one, greenish, etc.), but their brightness is much lower, making it difficult to quantitatively compare with the colors obtained on the very shiny wafers.

Comparison between the predicted color of the raw reflectance spectra with and without the top SiO_2_ layer, shown in Figure S1, confirmed that this layer does not have an influence on the color type, which is determined solely by the bottom SiO_2_ layer.

### Color Palette

**Figure 4:**
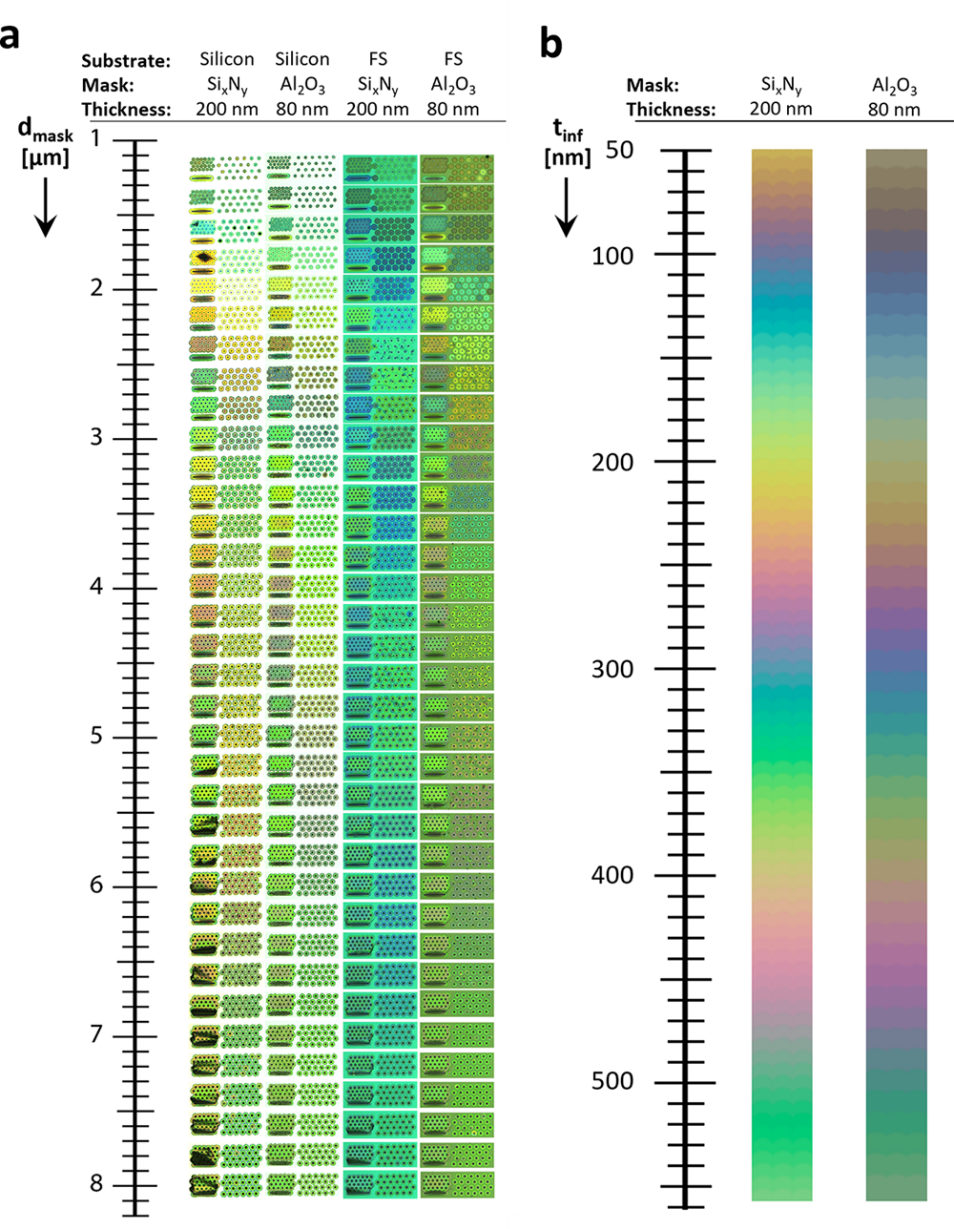
(a) Test structures comprising individual and merging arrays of holes of varying diameter *d*_mask_ and a straight line of constant height and width of half the nominal dimension. The structures were fabricated in all combinations of Al_2_O_3_ and Si_*x*_N_*y*_ as mask material on silicon and fused silica (FS) substrates. (b) Color generated by the spectra modeled as of the transfer matrix method for *t*_sup_ = 2700 nm, fixed *t*_mask_ indicated on the figure, and varying *t*_inf_ for masks made of Al_2_O_3_ and Si_*x*_N_*y*_. The color progression matches the experimentally observed colors.

Given the robustness of our methodology to predict and create different colors on the same wafer by adjusting microaperture sizes, we explored the potential to apply it to other substrates and different illumination modalities. Thus, we fabricated structures on silicon and fused silica substrates and with Al_2_O_3_ and Si_*x*_N_*y*_ as the mask with all the combinations of mask and substrate materials (four wafers in total). Since Figure 3a and b revealed that colors are more defined and saturated in the lower thickness range, the mask thickness was set to 80 nm for Al_2_O_3_ and 200 nm for Si_*x*_N_*y*_. The color palette simulated for both thicknesses and materials is represented in Figure 4b, validating that the created structural colors should be better defined and more saturated than the test samples. The fabrication process was optimized for each combination of materials, as described in detail in the Materials and Methods. Briefly, 80 nm of Al_2_O_3_ was deposited using atomic layer deposition (ALD) because this technique deposits films with a better thickness uniformity across the wafer than sputtering and Si_*x*_N_*y*_ was deposited using the LPCVD technique. The masks were etched through using ion beam etching for all material combinations. The under-etch of silicon substrates was performed in a vapor phase of XeF_2_ whose selectivity is infinite toward the Al_2_O_3_ mask and very high toward the Si_*x*_N_*y*_ mask. The under-etch of fused silica covered by an Al_2_O_3_ mask was performed using a vapor phase of HF with infinite selectivity, while the fused silica covered by Si_*x*_N_*y*_ was etched using a solution of 49% HF. The last step of LTO deposition was common for all four wafers.

The layout of each wafer is shown in Figure S2 and comprises test structure units with nominal dimensions ranging from 1 to 8 μm varied with an increment of 0.2 μm to obtain the full color range. Each unit is depicted in Figure S2 and comprises an array of holes of nominal diameter and spaced in order for the under-etched parts of the cavities to remain isolated (individual arrays), an array of holes of nominal diameter spaced so that the under-etched parts do merge (merging arrays) and a straight line with constant height and width of half a nominal dimension. The layout also contains visually appealing images of flowers, composed of six different parts, each made of an array of individual pixels with different diameters. Flowers of different lateral dimensions (height ranging from 0.6 to 20 mm) and aperture diameter combinations were patterned on the wafer.

The result of fabricating all the aforementioned test structures is shown in Figure 4a with the units arranged along *d*_mask_ to display the full color progression. The pictures were taken in identical conditions, varying only the intensity of the illumination and without applying image or color correction. Images of silicon substrates were set to saturate the background and leave the colorful units on a white background. This was not possible while imaging the fused silica substrates as the color pixels would saturate before the background. The colors were found to follow the same progression as shown in simulations, proving that the created structural colors can be controlled and predicted from the simulated parameters. However, the colors emerging from structures with Si_*x*_N_*y*_ masks were not found to be more saturated or defined than the other ones made from Al_2_O_3_ contrary to what was predicted by simulations. This difference can be attributed to the dielectric losses of silicon nitride that were not taken into account in the simulations. Furthermore, the color saturation was found to be different on fused silica and silicon substrates. Indeed the light reflected at the bottom of the cavity adds a constant component to the reflectance spectra whose signature is expected to be specific to the substrate material and its roughness.

Cracks in the suspended film originating from the edge of the line were observed in all cases, but these cracks were more pronounced in the cases with Si_*x*_N_*y*_ mask. In this latter configuration, the suspended structures created by the merging arrays were found to collapse, demonstrating a poorer mechanical stability. XeF_2_ etch rate was found to be limited by the opening in the mask and the lower range of *d*_mask_ had smaller underetch than the larger ones. Merging arrays had a brighter color thanks to the larger fill factor but did not have the same color as the individual arrays. The color in the middle of the array matched the color of individual arrays of larger diameter, indicating a larger *t*_inf_ for merging arrays than individual arrays. This can be explained by the fact that the bottom LTO layer benefits from multiple point sources for merging arrays. This explanation is supported by the observation of a different color on the edges of the merging arrays, especially visible in the larger range of *d*_mask_.

**Figure 5:**
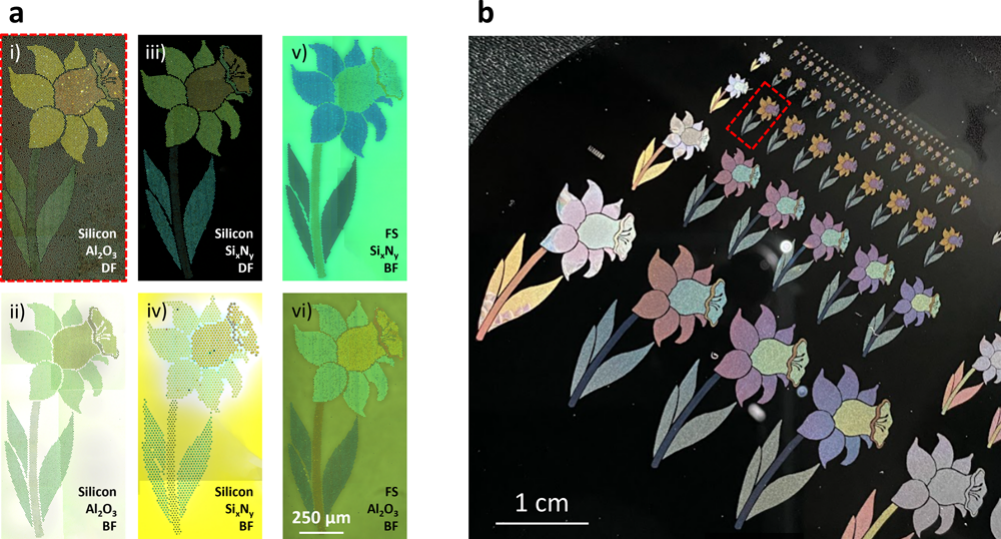
(a) Stitched microscopy images of colorful flowers fabricated in all combinations of Al_2_O_3_ (80 nm) and Si_*x*_N_*y*_ (200 nm) as mask material on silicon and fused silica (FS) substrates. Flowers fabricated on silicon substrates observed under dark field illumination (DF) revealed different colors than in bright field illumination (BF). (b) Picture of a silicon wafer with structures fabricated using 80 nm of Al_2_O_3_ as mask taken with a smartphone camera. The flower highlighted in red corresponds to the flower shown in (a) (i) revealing that the colors visible by eye match the colors observed in the dark field mode.

Finally, to demonstrate the large-scale capabilities of the proposed method of creating structural colors, we show in Figure 5 stitched optical microscopy images of colored flowers. The insets on each image indicate the substrate and mask materials, and whether the picture was taken in bright field (BF) or dark field (DF) conditions. The first observation is that the bright field and dark field colors do not match. Indeed the light in the dark field mode mainly comes from the substrate at a large angle, which considerably changes the interference spectrum. Second, the bright field image also comprises reflection from the substrate at the bottom of the cavity, which adds up to the interfering light from the top layers. The flowers made on fused silica substrates, however, did not display any color in the dark field mode (we attribute it to the fact that the light incident at a large angle was evenly diffused in the transparent substrate) and are thus not shown.

To further prove the real-world attractiveness of the proposed method, we show in Figure 5b a picture of the microfabricated flowers on the silicon wafer (with Al_2_O_3_ mask) taken with a standard smartphone camera using a white-light illumination from the side. The different structural colors are clearly visible by eye demonstrating the potential of the method to pattern arbitrary images from micrometer to centimeter scale. Notably, the first flower of each row on the left consist of merging arrays where the suspended membrane cracked and detached during the fabrication, explaining the absence of colors. Moreover, the colors observed by the eye (or by a smartphone camera) closely match those observed in the dark field mode under the microscope, which can be qualitatively seen from the microscope image of the flower shown in Figure 5a (i) and its image taken with a smartphone highlighted by the dashed red line in Figure 5b.

## Discussion

The spatial resolution of the proposed method is limited to the resolution of UV-photolithography (≈ 1 um structures), leading to a maximum DPI ≈ 25 000. This is comparable to the resolution of other published structural coloration methods,^22,32^ but an order of magnitude lower than the record-high DPI demonstrated using e-beam lithography or nanoimprinting.^10,33^ The calculated gamut values (Figure S3) for different configurations of the dielectric layers are below the typical values achieved using published all-dielectric structural color methods (maximum of 33.5% of the sRGB color space versus > 100% in literature^33^). This is a logical consequence of a multiple spectral peaks observed in the raw reflectance data (Figure S1), and their low absolute reflectance values. However, the easier fabrication process for the presented structural coloration method employing a single photomask can compensate for the loss in resolution and be a decisive factor for the attractiveness of the technique in large-scale commercial applications (especially in combination with imprinting techniques).

Given the current interest in dynamic structural color displays,^34^ three main potential solutions can be foreseen for such cavities. The cavities can be arranged to effectively form microchannels, in which a small amount of liquid can circulate.^35^ The color range can then be dynamically switched On and Off by circulating a drop of liquid matching the refractive index of the dielectric material of the apertures. The pixels under which the liquid is present then display the same color defined by *t*_mask_ only, canceling the effect of *t*_inf_ variations. A second method relies on the use of a material whose refractive index changes under an electric field such as lithium niobate or barium titanate as mask material. Application of an electric field via transparent electrodes made of ITO, for example, could change the color appearance up to a certain range. Similarly, a third method would employ a thermo-optic material to dynamically change the refractive index.

Importantly, given the size and fabrication method of such structures, when electric fields or heat is applied, one would need to consider potential effects on their thermal or piezo-electric expansion/shrinkage to ensure their mechanical stability. When using transparent substrates and explicitly considering the angle dependence in the calculation of the predicted spectrum (and color), such microapertures may be used similarly to other meta-surfaces^36^ to route specific colors to specific locations in transmission configuration for, e.g., sensing or imaging with pixelated detectors.

## Conclusion

In conclusion, we designed, tested, and optimized an interference-based method for creating structural color pixels from a stack of thin-film dielectrics. The method uses only a single UV photolithography step to define openings with desired diameters in the etch mask, which then define the reflected light wavelength via the varied aperture-controlled thickness of silicon dioxide layer deposited at the backside of the mask. The choice of both the substrate and the mask materials can be made such as to optimize the desired parameters of the reflected light. Being fully compatible with existing CMOS-oriented microfabrication facilities, such a method paves the way toward large scale applications of structural colors in industry, ranging from security features for anticounterfeiting to surface decoration in high-end watches and jewelry.

## Materials and methods

### Fabrication

All substrates were prepared for deposition by first performing the standard RCA cleaning.^37^

### Fused Silica with Al_2_O_3_ Mask

The test structures used for cross section characterization and reflectance measurements were fabricated by depositing a 500 nm thick Al_2_O_3_ layer using sputtering method (SPIDER 600, Pfeiffer). The structures fabricated for the color palette in Figure 4 start by depositing 80 nm of Al_2_O_3_ using atomic layer deposition (ALD) (TFS 200, Beneq). A 750 nm thick photoresist layer (AZ ECI 3007, MicroChemicals) was spin-coated and developed using an automated coater and developer (ACS200 GEN3, Süss) and exposed using direct writing methods (MLA150, Heidelberg Instruments). The pattern was transferred to the mask using ion beam etching (Nexus IBE350, Veeco) and the underetch performed by a vapor phase of HF (uEtch, SPTS) to lead to 5 μm lateral etching. Finally, a deposition of 2.7 μm of low temperature oxide (Centrotherm furnace) is performed to create the thin film stack.

### Fused Silica Substrate with Si_*x*_N_*y*_ Mask

200 nm of low stress Si_*x*_N_*y*_ was deposited using the LPCVD method (Centrotherm furnace). A 750 nm thick photoresist layer (AZ ECI 3007, MicroChemicals) was spin-coated and developed using an automated coater and developer (ACS200 GEN3, Süss) and exposed using direct writing methods (MLA150, Heidelberg Instruments). The pattern was transferred to the mask using ion beam etching (Nexus IBE350, Veeco). The underetch was then performed in a 49% solution of HF to lead to 5 μm lateral etching. Finally, a deposition of 2.7 μm of low temperature oxide (Centrotherm furnace) is performed to create the thin film stack.

### Silicon Substrate with Al_2_O_3_ Mask

80 nm of Al_2_O_3_ was deposited using atomic layer deposition (ALD) (TFS 200, Beneq). A 750 nm thick photoresist layer (AZ ECI 3007, MicroChemicals) was spin-coated and developed using an automated coater and developer (ACS200 GEN3, Süss) and exposed using direct writing methods (MLA150, Heidelberg Instruments). The pattern was transferred to the mask using ion beam etching (Nexus IBE350, Veeco) and the underetch performed by a vapor phase of XeF_2_ (Xactix, SPTS) to lead to 5 μm lateral etching for the largest dimensions. The etch rate was found to be aperture dependent and the smaller structures had a smaller underetch. Finally, a deposition of 2.7 μm of low temperature oxide (Centrotherm furnace) is performed to create the thin film stack.

### Silicon Substrate with Si_*x*_N_*y*_ Mask

200 nm of low stress Si_*x*_N_*y*_ was deposited using the LPCVD method (Centrotherm furnace). A 750 nm thick photoresist layer (AZ ECI 3007, MicroChemicals) was spin-coated and developed using an automated coater and developer (ACS200 GEN3, Süss) and exposed using direct writing methods (MLA150, Heidelberg Instruments). The pattern was transferred to the mask using ion beam etching (Nexus IBE350, Veeco) and the underetch performed by a vapor phase of XeF_2_ (Xactix, SPTS) to lead to 5 μm lateral etching for the largest dimensions. The etch rate was found to be aperture dependent and the smaller structures had a smaller underetch. Finally, a deposition of 2.7 μm of low temperature oxide (Centrotherm furnace) is performed to create the thin film stack.

### Reflectance Spectra Measurements

The experimental setup used for the spectral measurements is based on a commercial Olympus microscope used to focus a spot light to less than 5 μm diameter on the sample. The detailed description of the setup can be found elsewhere.^38^ The background spectrum (i.e., no illumination) was recorded and subtracted from every measure, see eq 9. The illuminant spectrum (i.e., the light source), used as a reference, was measured by placing a mirror at the sample plane, see eq 10. Each measurement on the samples was taken five times under the same conditions and the results were averaged, see eq 11. Overall, the reflectance spectrum of a sample is obtained as described by eq 12.
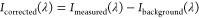
9
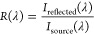
10
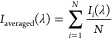
11
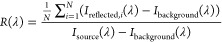
12

### Miscellaneous

The images of the samples were taken using a Leica DM8000 microscope, equipped with the DMC2900 camera, and acquired using the LAS X software set to provide no color correction. The layout was prepared using the AutoCAD software. The flower image was taken from the Web site reussiralecole.fr and used upon agreement with the owner. The flower layout was created by applying the *AND* logical operation between an array of holes of fixed diameter and a part of the flower. The calculation and modeling of the spectra and colors were made using custom Matlab 2022a code (available upon request).

## Supporting Information Available

Raw reflectance spectra of the test structures D1–D6 with theoretical spectra; layout of the structures depicted in Figure 4a; representation of the generated colors based on the calculated reflectance spectra on the CIE 1931 color space chromaticity diagram for masks of different thicknesses and materials (PDF)

## References

[ref1] Pastoureau M. Red: the History of a Color; Princeton University Press, 2017.

[ref2] Hooke R. Micrographia: Or Some Physiological Descriptions of Minute Bodies made by Magnifying Glasses with Observations and Inquiries thereupon; Jo. Martyn and J. Allestry, London, 1665. 10.5962/bhl.title.904

[ref3] Newton I. Red: the History of a Color; S. Smith and B. Walford, London, 1704.

[ref4] Frank F.; Ruska H. Übermikroskopische Untersuchung der Blaustruktur der Vogelfeder. Naturwissenschaften 1939, 27, 229–230. 10.1007/BF02716495.

[ref5] Fox H. M.; Vevers G. The Nature of Animal Colours; Sidgwick and Jackson, 1960.

[ref6] Zi J.; Yu X.; Li Y.; Hu X.; Xu C.; Wang X.; Liu X.; Fu R. Coloration Strategies in Peacock Feathers. Proc. Natl. Acad. Sci. U.S.A. 2003, 100, 12576–12578. 10.1073/pnas.2133313100. 14557541 PMC240659

[ref7] Xu T.; Shi H.; Wu Y.-K.; Kaplan A. F.; Ok J. G.; Guo L. J. Structural Colors: from Plasmonic to Carbon Nanostructures. Small 2011, 7, 3128–3136. 10.1002/smll.201101068. 21932283

[ref8] Duan H.; Hu H.; Kumar K.; Shen Z.; Yang J. K. Direct and Reliable Patterning of Plasmonic Nanostructures with Sub-10-nm Gaps. ACS Nano 2011, 5, 7593–7600. 10.1021/nn2025868. 21846105

[ref9] Kumar K.; Duan H.; Hegde R. S.; Koh S. C. W.; Wei J. N.; Yang J. K. W. Printing Colour at the Optical Diffraction Limit. Nat. Nanotechnol. 2012, 7, 557–561. 10.1038/nnano.2012.128. 22886173

[ref10] Zhu X.; Vannahme C.; Hojlund-Nielsen E.; Mortensen N. A.; Kristensen A. Plasmonic Colour Laser Printing. Nat. Nanotechnol. 2016, 11, 325–329. 10.1038/nnano.2015.285. 26657786

[ref11] Kristensen A.; Yang J. K. W.; Bozhevolnyi S. I.; Link S.; Nordlander P.; Halas N. J.; Mortensen N. A. Plasmonic Colour Generation. Nature Reviews Materials 2017, 2, 1–14. 10.1038/natrevmats.2016.88.

[ref12] Clausen J. S.; Højlund-Nielsen E.; Christiansen A. B.; Yazdi S.; Grajower M.; Taha H.; Levy U.; Kristensen A.; Mortensen N. A. Plasmonic Metasurfaces for Coloration of Plastic Consumer Products. Nano Lett. 2014, 14, 4499–4504. 10.1021/nl5014986. 25003515

[ref13] Li Z.; Clark A. W.; Cooper J. M. Dual Color Plasmonic Pixels Create a Polarization Controlled Nano Color Palette. ACS Nano 2016, 10, 492–498. 10.1021/acsnano.5b05411. 26631346

[ref14] Goh X. M.; Ng R. J. H.; Wang S.; Tan S. J.; Yang J. K. Comparative Study of Plasmonic Colors from All-Metal Structures of Posts and Pits. ACS photonics 2016, 3, 1000–1009. 10.1021/acsphotonics.6b00099.

[ref15] Chen F.; Wang S.-W.; Liu X.; Ji R.; Yu L.; Chen X.; Lu W. High Performance Colored Selective Absorbers for Architecturally Integrated Solar Applications. Journal of Materials Chemistry A 2015, 3, 7353–7360. 10.1039/C5TA00694E.

[ref16] Song M.; Li X.; Pu M.; Guo Y.; Liu K.; Yu H.; Ma X.; Luo X. Color Display and Encryption with a Plasmonic Polarizing Metamirror. Nanophotonics 2018, 7, 323–331. 10.1515/nanoph-2017-0062.

[ref17] Cui Y.; Hegde R. S.; Phang I. Y.; Lee H. K.; Ling X. Y. Encoding Molecular Information in Plasmonic Nanostructures for Anti-Counterfeiting Applications. Nanoscale 2014, 6, 282–288. 10.1039/C3NR04375D. 24189553

[ref18] Hong W.; Yuan Z.; Chen X. Structural Color Materials for Optical Anticounterfeiting. Small 2020, 16, 1907626 10.1002/smll.201907626. 32187853

[ref19] Li Z.; Dai Q.; Deng L.; Zheng G.; Li G. Structural-Color Nanoprinting with Hidden Watermarks. Opt. Lett. 2021, 46, 480–483. 10.1364/OL.417026. 33528389

[ref20] Lapidas V.; Zhizhchenko A.; Pustovalov E.; Storozhenko D.; Kuchmizhak A. A. Direct Laser Printing of High-Resolution Physically Unclonable Function Anti-Counterfeit Labels. Appl. Phys. Lett. 2022, 120, 261104 10.1063/5.0091213.

[ref21] Proust J.; Bedu F.; Gallas B.; Ozerov I.; Bonod N. All-Dielectric Colored Metasurfaces with Silicon Mie Resonators. ACS Nano 2016, 10, 7761–7767. 10.1021/acsnano.6b03207. 27458790

[ref22] Sun S.; Zhou Z.; Zhang C.; Gao Y.; Duan Z.; Xiao S.; Song Q. All-Dielectric Full-Color Printing with TiO2Metasurfaces. ACS Nano 2017, 11, 4445–52. 10.1021/acsnano.7b00415. 28317376

[ref23] Flauraud V.; Reyes M.; Paniagua-Domínguez R.; Kuznetsov A. I.; Brugger J. Silicon Nanostructures for Bright Field Full Color Prints. ACS Photonics 2017, 4, 1913–1919. 10.1021/acsphotonics.6b01021.

[ref24] Yang J. H.; Babicheva V. E.; Yu M. W.; Lu T.-C.; Lin T.-R.; Chen K.-P. Structural Colors Enabled by Lattice Resonance on Silicon Nitride Metasurfaces. ACS Nano 2020, 14, 5678–5685. 10.1021/acsnano.0c00185. 32298575

[ref25] Li H.; Xu Y.; Zhang X.; Xiao X.; Zhou F.; Zhang Z. All-Dielectric High Saturation Structural Colors Enhanced by Multipolar Modulated Metasurfaces. Opt. Express 2022, 30, 28954–28965. 10.1364/OE.464782. 36299081

[ref26] Daqiqeh Rezaei S.; Dong Z.; You En Chan J.; Trisno J.; Ng R. J. H.; Ruan Q.; Qiu C.-W.; Mortensen N. A.; Yang J. K.W. Nanophotonic Structural Colors. ACS Photonics 2021, 8, 18–33. 10.1021/acsphotonics.0c00947.

[ref27] Xuan Z.; Li J.; Liu Q.; Yi F.; Wang S.; Lu W. Artificial Structural Colors and Applications. Innovation-the European Journal of Social Science Research 2021, 2, 100081 10.1016/j.xinn.2021.100081. PMC8454771 34557736

[ref28] Macleod H. A.; Macleod H. A. Thin-Film Optical Filters; CRC Press, 2010.

[ref29] Cheng L.-Y.; McVittie J. P.; Saraswat K. C. New Test Structure to Identify Step Coverage Mechanisms in Chemical Vapor Deposition of Silicon Dioxide. Appl. Phys. Lett. 1991, 58, 2147–2149. 10.1063/1.104988.

[ref30] Wille H.; Burte E.; Ryssel H. Simulation of the Step Coverage for Chemical Vapor Deposited Silicon Dioxide. J. Appl. Phys. 1992, 71, 3532–3537. 10.1063/1.350908.

[ref31] Pedrotti F. L.; Pedrotti L. S. Introduction to Optics; Prentice Hall, 1996; Chapter 19.

[ref32] Yang Z.; Chen Y.; Zhou Y.; Wang Y.; Dai P.; Zhu X.; Duan H. Microscopic Interference Full-Color Printing Using Grayscale-Patterned Fabry-Perot Resonance Cavities. Advanced Optical Materials 2017, 5, 1700029 10.1002/adom.201700029.

[ref33] Dong Z.; Ho J.; Yu Y. F.; Fu Y. H.; Paniagua-Dominguez R.; Wang S.; Kuznetsov A. I.; Yang J. K. Printing beyond sRGB color gamut by mimicking silicon nanostructures in free-space. Nano Lett. 2017, 17, 7620–7628. 10.1021/acs.nanolett.7b03613. 29115134

[ref34] Wu Y.; Chen Y.; Song Q.; Xiao S. Dynamic Structural Colors Based on All-Dielectric Mie Resonators. Advanced Optical Materials 2021, 9, 2002126 10.1002/adom.202002126.

[ref35] Lipp C.; Uning K.; Cottet J.; Migliozzi D.; Bertsch A.; Renaud P. Planar hydrodynamic traps and buried channels for bead and cell trapping and releasing. Lab Chip 2021, 21, 3686–3694. 10.1039/D1LC00463H. 34518854 PMC8477447

[ref36] Chen B. H.; Wu P. C.; Su V.-C.; Lai Y.-C.; Chu C. H.; Lee I. C.; Chen J.-W.; Chen Y. H.; Lan Y.-C.; Kuan C.-H.; et al. GaN metalens for pixel-level full-color routing at visible light. Nano Lett. 2017, 17, 6345–6352. 10.1021/acs.nanolett.7b03135. 28892632

[ref37] Kern W. The Evolution of Silicon Wafer Cleaning Technology. J. Electrochem. Soc. 1990, 137, 1887 10.1149/1.2086825.

[ref38] Ray D.; Wang H.-C.; Kim J.; Santschi C.; Martin O. J. F. A Low-Temperature Annealing Method for Alloy Nanostructures and Metasurfaces: Unlocking a Novel Degree of Freedom. Adv. Mater. 2022, 34, 2108225 10.1002/adma.202108225. 35167722

