# Supplementary material for: Aperture-Controlled Fabrication of All-Dielectric Structural Color Pixels
Source: ACS Appl Mater Interfaces. 2023 Jun 29;15(27):33056–64. doi: 10.1021/acsami.3c03353 (PMC10347118; doi:10.1021/acsami.3c03353)
Supplement: Supplementary file 1 — am3c03353_si_001.pdf [file am3c03353_si_001.pdf]

Supplementary Material for
Aperture-controlled fabrication of all-dielectric structural color pixels

Clémentine Lipp,† Audrey Jacquillat,† Daniel Migliozzi,† Hsiang-Chu Wang,‡ Arnaud Bertsch,† Evgenii Glushkov,† Olivier J.F. Martin‡ and Philippe Renaud∗†

†Ecole Polytechnique Fédérale de Lausanne EPFL-STI-IMT-LMIS4, Station 17, CH-1015 Lausanne, Switzerland

‡Ecole Polytechnique Fédérale de Lausanne EPFL-STI-IMT-NAM, Station 11, CH-1015 Lausanne, Switzerland
*E-mail: philippe.renaud@epfl.ch

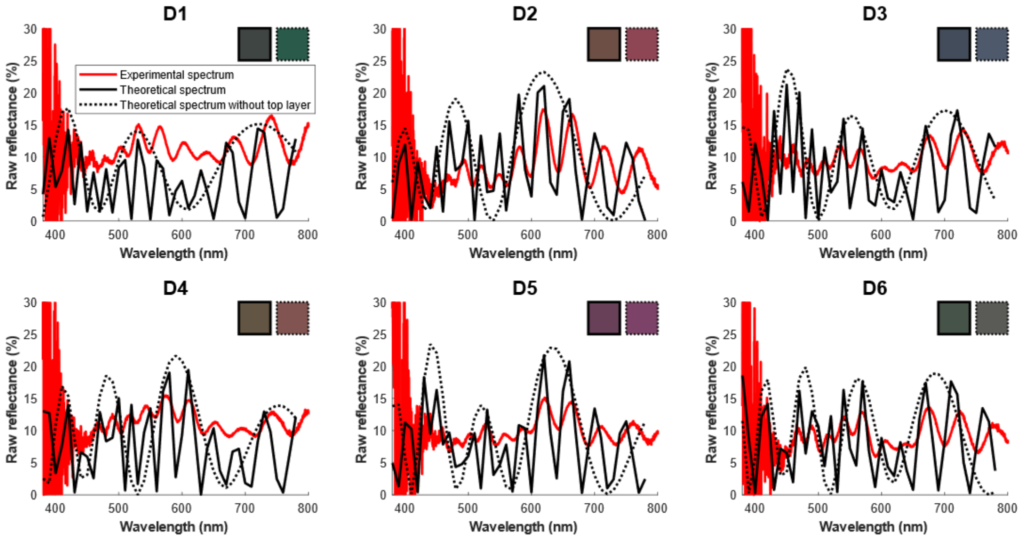

Figure S1 Raw reflectance spectra of the test structures represented in Figure 2**b.** The theoretical spectra with and without top LTO layer are also represented with the corresponding rendered color.

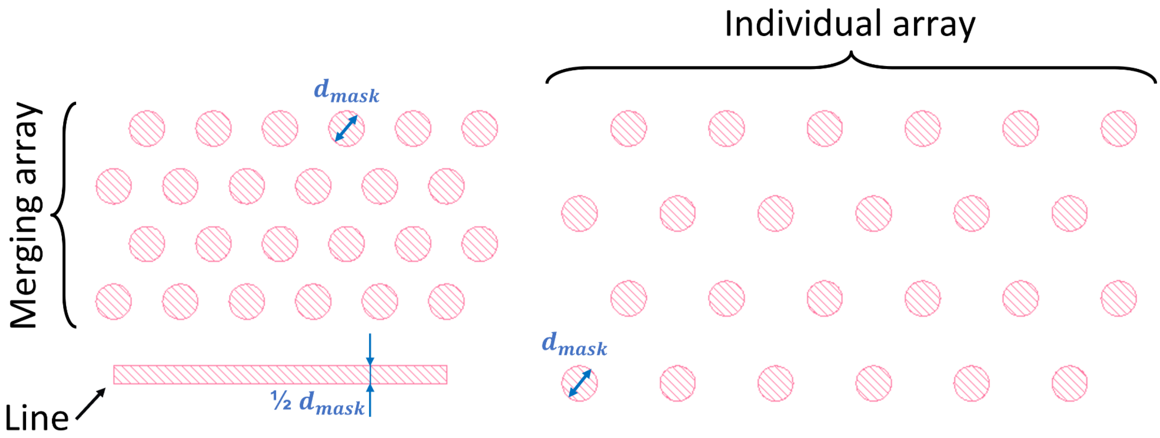

Figure S2 Layout of the test structures fabricated with different mask material and onto different substrate types shown in Figure 4**a.**

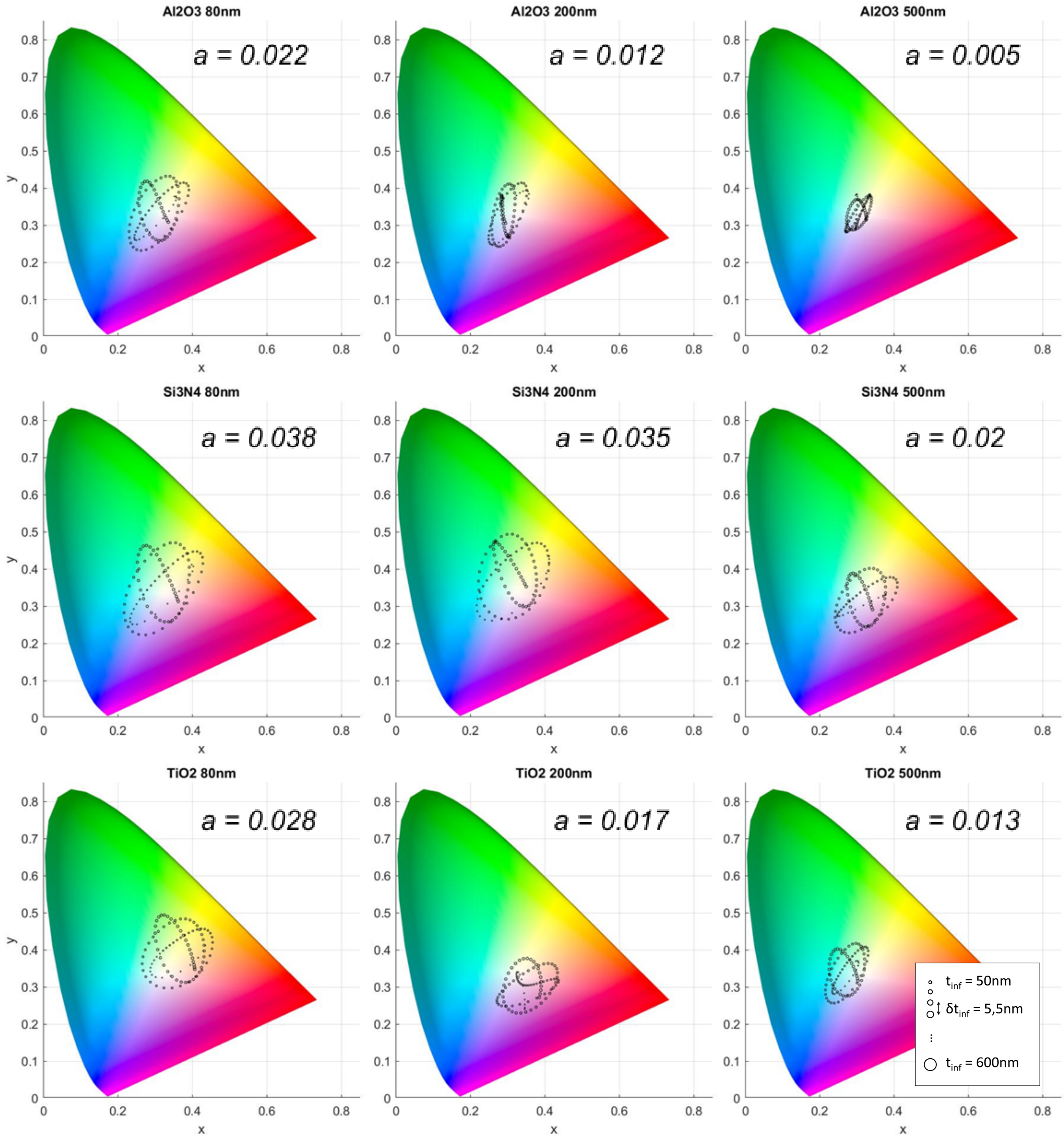

Figure S3 Representation of the generated colors based on the calculated reflectance spectra on the CIE 1931 color space chromaticity diagram for masks of different thicknesses (80, 200 and 500nm) and materials (Al_2O_3, Si_xN_y and TiO_2) and t_{inf} progressively increasing from 50 nm to 600 nm with steps of 5.5 nm with the surface area *a* covered by each configuration indicated on the plots.
